# A comprehensive transcriptome analysis of skeletal muscles in two Polish pig breeds differing in fat and meat quality traits

**DOI:** 10.1590/1678-4685-GMB-2016-0101

**Published:** 2018

**Authors:** Katarzyna Piórkowska, Kacper Żukowski, Katarzyna Ropka-Molik, Mirosław Tyra, Artur Gurgul

**Affiliations:** 1 Department of Animal Molecular Biology, National Research Institute of Animal Production, Balice, Poland; 2 Department of Pig Breeding, National Research Institute of Animal Production, Balice, Poland; 3 Department of Cattle Breeding, National Research Institute of Animal Production, Balice, Poland

**Keywords:** RNA-seq, firmness, fat content, Polish pigs

## Abstract

Pork is the most popular meat in the world. Unfortunately, the selection pressure focused on high meat content led to a reduction in pork quality. The present study used RNA-seq technology to identify metabolic process genes related to pork quality traits and fat deposition. Differentially expressed genes (DEGs) were identified between pigs of Pulawska and Polish Landrace breeds for two the most important muscles (*semimembranosus* and *longissimus dorsi*). A total of 71 significant DEGs were reported: 15 for *longissimus dorsi* and 56 for *semimembranosus* muscles. The genes overexpressed in Pulawska pigs were involved in lipid metabolism (*APOD*, *LXRA, LIPE, AP2B1, ENSSSCG00000028753* and *OAS2*) and proteolysis (*CST6, CTSD, ISG15* and *UCHL1*). In Polish Landrace pigs, genes playing a role in biological adhesion (*KIT, VCAN, HES1, SFRP2, CDH11, SSX2IP* and *PCDH17*), actin cytoskeletal organisation (*FRMD6, LIMK1, KIF23* and *CNN1*) and calcium ion binding (*PVALB, CIB2, PCDH17, VCAN* and *CDH11*) were transcriptionally more active. The present study allows for better understanding of the physiological processes associated with lipid metabolism and muscle fiber organization. This information could be helpful in further research aiming to estimate the genetic markers.

## Introduction

Over the past few decades, meat content of pig carcasses has been significantly increased. The intention was to decrease backfat thickness, but unfortunately level of intramuscular fat (IMF) was also reduced. The breeding efforts were motivated by societal needs, especially in Poland, where a high meat yield was the aim. Today, Polish pigs have a thin backfat, low percentage of IMF and poor texture parameters, which influence an inferior flavour and low technological suitability (Tyra and Zak, 2013). The traditional breeding methods are expensive and time-consuming because they are based on an evaluation of adult pigs. Therefore, the development of genetic markers associated with the desired phenotype should indicate animals having breeding potential.

To date, several interesting discoveries are made in the field of gene polymorphisms affecting pork quality. When analyzing intramuscular fat (IMF), which is considered as the main factor determining the flavour of meat, it was observed that mutations in *H-FABP*, (Pang *et al.*, 2006), *LPL* and *LIPE* genes (Xue *et al.*, 2015) influenced IMF content. In turn, Zhang *et al.* (2014) evaluating the effect of 33 single nucleotide polymorphisms (SNPs) on pork quality traits showed that mutations in *AMPD1*, *ADIPOQ* and *COP1* were associated with juiciness, and *FTO, TNF, HSP70.2* and *CAST* were involved in pork color determination. The *CAST* gene encodes calpastatin, a key enzyme in the calpain system. In a study by Ropka-Molik *et al.* (2014a), it was established that *CAST* also affected meat texture parameters, such as firmness and toughness, as well as pH and water holding capacity (WHC).

Pork tenderness was considered by Miller *et al.* (2001) as the most important factor determining technological suitability. This parameter is associated with post-mortem tenderisation, when the proteolysis process is activated in response to a reduction of pH, including the calpain system responsible for the conversion of muscle to meat, the caspase cascade associated with apoptosis, and also cathepsin proteases produced by lysosomes (Huff- Lonergan *et al.*, 2010). The genes encoding these proteins were considered as potential candidates for pork quality traits, but without spectacular discoveries.

Currently, the estimation of genetic markers for farm animal quantitative traits represents a major challenge. In pigs, only several genes (*RYR1, PRKAG3* and *IGF2*) with major phenotypic effect were identified. Because the quantitative traits are the result of the cooperation of many genes, capturing the function and interaction of the whole genome is necessary. A new evaluation method of dairy cattle, the genomic estimated breeding value (GEBV), was developed (Hayes *et al.*, 2009). The method uses SNP microarray results as support for the traditional breeding evaluation.

On of the genomics methods, RNA-seq, is used to analyze transcriptome profiles. This method has been widely applied in recent years because the RNA-seq results are much more informative in comparison to the results obtained by gene expression microarray technology. The microarray technique is unable to detect new transcripts, gene translocation, inversion and alternative splice variants (Hurd and Nelson, 2009), whilst RNA-seq provides these opportunities. The major application of the RNA-seq method is an evaluation of differentially expressed genes (DEGs) between investigated groups. For example in pigs, the RNA-seq method was used to estimate the transcriptome profile depending on breed (Ropka-Molik *et al.*, 2014b), type of tissue (Esteve-Codina *et al.*, 2011) and phenotype (Corominas *et al.*, 2013). The RNA-seq method also provides information on the transcript sequences, and therefore could be used for identification of gene mutations. Martínez-Montes *et al.* (2016) detected potential genetic markers for porcine growth and fat traits using RNA-seq.

In the present study, the comparison of muscle transcriptomic profiles between Pulawska (PUL) and Polish Landrace (PL) breeds was performed. The investigated pig groups showed highly significant differences in fat content of the carcass and in meat quality traits. PUL is one of the indigenous Polish breeds included in the genetic resources conservation programme (Szyndler-Nedza *et al.*, 2010) that was not under selection pressure. Therefore, PUL pork characterizes with high meat quality, fat content, and is recognized as a delicacy on Polish tables (Kasprzyk *et al.*, 2015). A few years ago, it was proposed to use the ham of these pigs as a dry-cured product (Olkiewicz, 2009). In numerous countries, indigenous pigs are maintained as a genetic diversity reservoir. They are usually characterized by high-fat content and very good reproduction performance, such as the Chinese Tongcheng pigs (Fan *et al.*, 2006), and by low growth rate as in the Zimbabwe Mukota pigs (Chimonyo *et al.*, 2010). The second investigated PL breed is used in Polish breeding as a maternal component. These pigs characterize with good reproductive performance, high meat content, and growth traits. Nevertheless, as a result of the breeding efforts, PL pork is not as tasty, due to high drip loss and low IMF content (Tyra and Zak, 2013). PL shows high similarity to other white pigs maintained in Europe, because it originated from the Swedish Landrace and the German Large White.

Consequently, the present study has attempted to identify genes involved in the determination of pork traits, including the regulation of fat metabolism, meat quality and growth performance.

## Material and Methods

### Animals

The study was conducted on 16 gilts of PUL (n=8) and PL (n=8). The animals were maintained at the Pig Testing Station of the National Research Institute of Animal Production in Chorzelów under the same housing and feeding conditions. The pigs came from different farms and were unrelated. They were delivered to the test station as piglets and fed *ad libitum* from 30 up to 100 (± 2.5) kg of body weight, after which they were starved for 24 h before slaughter. Stunning with high-voltage electric tongs was followed by exsanguination. After chilling for 24 h at 4 °C, the right half-carcass was evaluated. Carcass traits were measured according to Tyra and Zak (2013). Meat texture parameters and pH for the *longissimus dorsi* (LD) and *semimembranosus* (S) muscles were determined according to Ropka-Molik *et al.* (2014a). The meat exudation, IMF and meat colour were measured in *longissimus dorsi*. The meat exudation was determined as the amount of free water according to the filter paper press method of Grau and Hamm (1953) as the ratio between pressed water (meat exudate) to total water content, where 1 cm^2^ of expressed juice ring after pressing corresponds to 10 mg of water loss, and total water content is 75% of the total meat weight (Huff-Lonergan and Lonergan, 2005). IMF and meat color were measured as described by Tyra *et al.* (2013). Muscle samples for molecular analysis were collected immediately (up to 20 min) after slaughter, stabilized in RNAlater solution (Ambion) and stored at -20 °C.

### NGS library construction

RNA was isolated using TRI Reagent (Applied Biosystems) according to the manufacturer’s protocol. Muscle samples were homogenized using a Bullet Blender 24 homogenizer (Next Advance). The RNA (Agencourt RNAClean XP kit) was purified by a bead method and its quality and quantity were assessed fluoremetrically (Qubit Fluorometer, Invitrogen) and by the TapeStation 2200 system (RNA tapes, Agilent). RNA integrity number (RIN) was in the range between 6.8-8. Ribosomal RNA from 5000 ng of total RNA was removed using a Ribo-Zero Gold rRNA Removal Kit (Human/Mouse/Rat) (Epicentre). The absence of rRNA was verified on the TapeStation 2200 system. The elimination of rRNA leads to a flattening of the differences in transcript levels between the groups and allows for sequencing of cDNA libraries presenting low frequency (Benes *et al.*, 2011). A TruSeq RNA Sample Preparation Kit v2 (Illumina) was used to prepare cDNA libraries from 100 ng aliquots of rRNA-depleted samples according to the manufacturer’s protocol. The cDNA samples were ligated with indexed adaptors in the order shown in Table S1. The libraries were amplified in 15 cycles of PCR, and their quantity was estimated using the Qubit 2.0 Fluorometer and 2200 TapeStation (D1000 tapes). The final concentration of the cDNA libraries was normalized to 10 nM, after which the libraries were pooled (Table S1).

### Whole genome sequencing

The libraries were diluted according to a cluster generation protocol and loaded into a v3 Illumina Flowcell (16 samples per line with four technical replicates; the technical replicates were used to avoid the lane effect). Single-read clusters were generated on a cBot system (Illumina). Flowcell clustering was performed using TruSeq SR Cluster Kit v3-cBot- 4 HS. Sequencing-by-synthesis of the clustered libraries was conducted on a HiScanSQ System in 81 bp single-end cycles using TruSeq SBS Kit v3-HS chemistry (Illumina).

### Raw reads processing

The raw sequences were qualitatively controled using the FastQC tool. Next, Flexbar software was used to remove Illumina adapters, poly-A sequences and reads shorter than 36 bp or with a quality score<20. Filtered sequences were aligned to the *Sus scrofa* genome (Sscrofa10.2 assembly) with reference annotation containing the 21,630 genes listed in the Ensembl database. Alignment and estimation of the gene expression levels were performed using the RSEM package supported by Bowtie 2 aligner. The alignment and DEG statistics were generated using the Picard tools (http://picard.sourceforge.net and RNA-SeQC tools). The sequence data were submitted to Gene Expression Omnibus (accession no. GSE75707, https://www.ncbi.nlm.nih.gov/geo/query/acc.cgi?acc=GSE75707).

### DEG analysis

The DEG analysis was performed using edgeR (Robinson and Oshlack, 2010), baySeq (Hardcastle and Kelly, 2010) and DESeq2 (Love *et al.*, 2014) separately for each muscle and breed, which was preceded by PCA (DESeq2) and MDS (edgeR) analyses. The significance for particular methods was presented as false recovery rate (FDR) by edgeR and baySeq, and as adjusted *P*-values by DESeq2. Transcripts with a fold change ≥ 1.30 and evaluated by at least two methods as significant (*P* ≤ 0.05), were included in the further analysis. Functional analysis of DEGs was performed by Panther Gene List software, where the *P*-value was estimated in overrepresentation tests and Kobas 3.0, which predicts biological pathways based on the KEGG, Reactome and Biocyc databases.

### Gene panel validation by qPCR

Seventeen differentially expressed genes were selected for qPCR validation: *CNN1, PVALB, HP, OAS1, LXRA, APOD, LIMK1, PEX11G*, *UCHL1, MAOB*, *ASS1, GPX3, VCAN*, *SFRP2, HES1, PPP1R11, LIPE.* Primers and probes for the investigated genes were purchased from Applied Biosystems as TaqMan Assays (Applied Biosystems), or primers for some genes were designed in Primer3 and synthesized by Genomed (Poland). The primers and assays used in the validation were presented in Table S2. cDNA was prepared from 1,000 ng of total RNA by using TRANSCRIPTME Reverse Transcriptase (DNA Gdansk). TaqMan® Gene Expression Master Mix (Applied Biosystem) and AmpliQ 5x Hot EvaGreen (Novazym) were used to perform analyses on a 7500 Real-Time PCR System (Applied Biosystems). Each qPCR analysis was performed with three replicates. Mean CT values were obtained with an acceptable error of 0.3. Relative mRNA abundance (RQ) was measured by the ΔΔCT method. As an endogenous control, two genes, *OAZ1* and *RPL27,* were used. These are known as being stable in regard to the expression in muscle tissue. The M values describing the stability of endogenous control genes were estimated for *OAZ1* and *RPL27* by geNorm, and they were under 0.5. The comparison between RNA-seq and qPCR results was performed using Pearson’s correlation analysis, and significant differences in gene expression (qPCR) between analyzed groups were estimated using ANOVA with post-hoc Duncan test (SAS Enterprise).

### Correlation analysis

Seventeen validated genes were included in the Pearson’s correlation (SAS Enterprise) analysis for important growth, carcass and meat quality traits with *P*-value thresholds: *P≤0.05, **P ≤0.01, ***P≤0.001, and also the Bonferroni corrected P-value. The comparison between porcine traits and FPKM was performed for each muscle separately. For the most interesting association, regression plots were created.

## Results

### Animal characteristics

In the present study, 16 gilts of two pig breeds were evaluated in regard to carcass and meat quality parameters. The PUL backfat was thicker by 40% (*P*<0.05). In turn, the investigated PL pigs were characterized by higher ham mass, daily gain by 210 g (*P*<0.01), meat percentage and better feed conversion (*P*<0.01). Moreover, as was expected, PL pork was less tender and had higher water exudation than that of PUL pigs (Table 1), which was observed for both analyzed muscles.

**Table 1:** Characteristics of the pig, breeds, means ± SD.

Traits	PL (n=8)		PUL (n=8)	
	Mean	SD	Mean	SD
Daily gain (g)	972^A^	86.6	763^B^	48.2
Feed conversion (kg/kg)	2.60^A^	0.08	2.96^B^	0.14
Carcass yield %	76.24	2.27	75.64	3.98
Weight of loin (kg)	6.19	0.57	5.65	0.50
Weight of ham (kg)	9.31^a^	0.26	8.63^b^	0.74
Backfat thickness (cm)	1.18^a^	0.17	1.64^b^	0.40
Loin eye area (cm^2^)	52.50	5.25	48.94	5.18
Meat percentage %	63.04^a^	1.95	59.5 ^b^	2.80
*Longissimus dorsi* muscle				
Meat exudation	41.50^A^	5.11	28.32^B^	1.24
Meat color				
Meat lightness	55.08	2.21	53.72	1.74
Meat redness	16.70	1.19	16.16	0.81
Meat yellowness	2.16	0.99	2.25	0.62
Intramuscular fat	1.08	0.21	1.19	0.12
pH45	6.33	0.18	6.32	0.19
pH24	5.63	0.05	5.60	0.06
Firmness by WB (cooked meat)	122.58^A^	14.7	57.7^B^	3.21
Toughness by WB (cooked meat)	277.07^A^	37.6	140.97^B^	14.2
Harness by TPA (cooked loin)	6.95	2.7	5.01	2.05
*Semimembranosus* muscle				
pH45	6.30	0.12	6.26	0.16
pH24	5.64	0.04	5.62	0.05
Firmness by WB (cooked meat)	89.92^a^	10.17	75.13^b^	6.83
Toughness by WB (cooked meat)	203.91	39.72	180.30	35.86
Harness by TPA (cooked meat)	9.30	3.84	8.25	3.88

Abbreviation: SD– standard deviation, TPA–texture parameter analysis, WB– Warner-Bratzler, PL– Polish Landrace, PUL– Pulawska. Values with the same superscripts show significant differences between genotypes (A, B = *P*<0.01. a, b = *P*<0.05)

### Transcriptome analysis

The average number of raw reads detected per sample was 23,633,127, and after filtration it was 23,453,870. After mapping to the pig reference genome (Sscrofa10.2 assembly) (Table S1), 63.15% of the reads matched annotated exon regions, and 7.83% matched introns. The comparison of transcriptome profiles between the analyzed breeds showed that in the PUL *longissimus dorsi* (LD) muscle, six overexpressed genes were detected, and in *semimembranosus* (S), 28 genes showed increased expression (Tables 2 and S3). Among these up-regulated genes, there are some engaged in lipid metabolism (*ENSSSCG00000028753, LIPE, LXRA, APOD, GPX3* and *AP2B1*), actin filament building (*LIMK1*), proteolysis (*CTSD, CST6*, *UCHL1, ISG15, LXRA*), and carbohydrate derivative binding *(KHK, SEPT6, LIMK1, ASS1, HP, OAS2*) (Tables 3 and 4). In turn, genes overexpressed in PL muscles are involved in several biological processes, such as cell adhesion (*KIT, VCAN, HES1, SFRP2, CDH11, SSX2IP, PCDH17*), calcium ion binding (*PVALB, CIB2, PCDH17, VCAN* and *CDH11*), and actin organisation (*CNN1, FRMD6, KIF23*) (Tables 3 and 4). The differentially expressed isoform analysis showed that in both muscles of PUL the seventh isoform of the *SEPT6* gene showed increased expression. This isoform is composed of 10 exons, which encode all three important protein domains.

**Table 2:** Genes and isoforms ≥ ± 1.5-fold (up-regulated and down-regulated), differentially expressed in *longissimus dorsi* muscle of Polish Landrace pigs.

Gene		Ensembl ID	DESeq		edgeR		baySeq FDR	Validation qPCR PC^1^	
								*r*	*P*
Symbol	Name		FC	Ad *P*-value	FC	FDR			
*PPP1R11*	Protein phosphatase 1, regulatory (inhibitor) subunit 11	*ENSSSCG00000028347*	2.40	***	5.13	***	***	0.63	≤0.05
*CNN1*	calponin 1, basic, smooth muscle	*ENSSSCG00000013614*	2.06	***	2.60	***	***	0.89	≤0.001
*NG*	Similar to NADH dehydrogenase (ubiquinone) 1 alpha subcomplex, 6, (*NDUFA6*)	*ENSSSCG00000021119*	2.05	*	4.46	**	***		
*PVALB*	Parvalbumin	*ENSSSCG00000000138*	1.91	*	4.26	*	*	0.97	≤0.001
*NG*	Similar to transmembrane protein 106C (*TMEM106C*)	*ENSSSCG00000028397*	1.88	*	2.75	*	0.057		
*CIB2*	Calcium and integrin binding family member 2	*ENSSSCG00000001762*	1.81	**	2.12	*	***		
*CHPT1*	Cholinephosphotransferase 1	*ENSSSCG00000000864*	1.66	*	1.86	0.051	*		
*TFDP2*	Transcription factor Dp-2	*ENSSSCG00000026809*	1.58	**	1.66	0.17	**		
*UBE2J1*	Ubiquitin-conjugating enzyme E2 J1	*ENSSSCG00000004317*	1.55	*	1.62	0.254	*		
*IGLC*	Ig lambda chain C region	*ENSSSCG00000010044*	-1.48	0.321	-8.06	*	***		
*BCAR3*	Breast cancer anti-estrogen resistance protein 3	*ENSSSCG00000006893*	-1.80	**	-2.09	0.055	**		
*OAS2*	2’-5’-oligoadenylate synthase 2	*ENSSSCG00000009881*	-1.91	*	-3.04	*	*	0.91	≤ 0.001
*HP*	Haptoglobin	*ENSSSCG00000002749*	-1.95	*	-5.34	*	***	0.92	≤ 0.001
*NG*	Similar to cytochrome P450 family 4 subfamily F member 3hydroxylase 1 (*CYP4F3 or CYP4F55)*	*ENSSSCG00000028753*	-1.96	*	-3.11	*	***		
*SEPT6-007*	6-isoform of septin 6	*ENSSSCT00000013798*	-1.76	*	-2.11	*	*		

Abbreviation: Ad *P*–value -adjusted *P*-value, FDR– false discovery rate; *P*-value: ***P<0.001, **P<0.01, *P<0.05, FC– fold change, PL– Polish Landrace pigs, NG– novel gene according to Ensembl browser,

1 Pearson correlation coefficient r

**Table 3:** Functional annotation of differentially expressed genes in *longissimus dorsi* muscle of pigs.

Gene ontology	Genes			
	*P*-value	Corrected P-value	up-regulated in Pulawska pigs	up-regulated in Polish Landrace
GO:0072562 blood microparticle	1.62E-05	1.20E-02	*HP, IGLC*	*CIB2*
GO:0032515 negative regulation of catabolic process	1.61E-03	1.16E-01	*HP,*	*UBE2J1*
GO:0018377 protein myristoylation	2.62E-03	1.16E-01	*OAS2*	
GO:1901568 fatty acid derivative metabolic process	4.80E-03	1.16E-01	*ENSSSCG00000028753*	
GO:0019992 diacylglycerol binding	5.24E-03	1.16E-01		*CHPT1*
GO:0051480 regulation of cytosolic calcium ion concentration	5.90E-03	1.16E-01		*CIB2, PVALB*
GO:0009056 catabolic process	6.99E-03	1.16E-01	*HP, ENSSSCG00000028753, OAS2*	*UBE2J1*
GO:0009101 glycoprotein biosynthetic process	9.32E-03	1.20E-01		*CIB2*
GO:0005924 cell-substrate adherens junction	3.11E-03	1.21E-01		*CIB2, CNN1*
GO:0061631 ubiquitin conjugating enzyme activity	1.26E-02	1.21E-01		*UBE2J1*
GO:0031594 neuromuscular junction	7.89E-03	1.21E-01		*CIB2*
GO:0042158 lipoprotein biosynthetic process	3.83E-02	1.48E-01	*OAS2*	
GO:0033559 unsaturated fatty acid metabolic process	4.58E-02	1.48E-01	*ENSSSCG00000028753*	
GO:0005178 integrin binding	1.73E-02	1.24E-01		*UBE2J1*
GO:0042758 long-chain fatty acid catabolic process	3.67E-03	1.48E-01	*ENSSSCG00000028753*	
GO:0005509 calcium ion binding	9.71E-03	1.21E-01	*ENSSSCG00000028753*	
GO:0042383 sarcolemma	1.65E-02	1.24E-01		*CIB2*
GO:0031032 actomyosin structure organization	2.43E-02	1.24E-01		*CNN1*
Pathway				
R-SSC-1483206 glycerophospholipid biosynthesis	4.75E-02	1.50E-01		*CHPT1*
PWY3O-450 phosphatidylcholine biosynthesis	3.06E-03	1.16E-01		*CHPT1*
R-SSC-211935 fatty acids	6.98E-03	1.16E-01	*ENSSSCG00000028753*	
R-SSC-2142753 arachidonic acid metabolism	2.42E-02	1.34E-01	*ENSSSCG00000028753*	
R-SSC-556833 metabolism of lipids and lipoproteins	3.78E-02	1.48E-01	*ENSSSCG00000028753*	*CHPT1*
PWY-7511 protein ubiquitylation	1.82E-02	1.27E-01		*UBE2J1*
ssc00565 ether lipid metabolism	2.70E-02	1.29E-01		*CHPT1*

GO-gene ontology and pathways were estimated by Kobas 3.0 and Panther Gene List

**Table 4:** Functional annotation of differentially expressed genes in *semimembranosus* muscle of pigs.

Gene ontology	*P*-value	Corrected *P*-value	Genes	
			up-regulated in Pulawska	up-regulated in Polish Landrace
Gene ontology				
GO:0007018 microtubule-based movement	8.43E-06	4.23E-03	*AP2B1, UCHL1*	*TTC21B, DNAH11, KIF23, SSX2IP*
GO:0032989 cellular component morphogenesis	1.21E-05	4.81E-03	*LIMK1, UCHL1,*	*ENSSSCG00000016843, HES1, SFRP2, TTC21B, FRMD6, TCHP, CDH11, NR4A2, SSX2IP*
GO:0009056 catabolic process	3.62E-05	7.78E-03	*CTSD, RGP1, HSL, UCHL1, HP, GPX3, OAS2, MAOB, KHK, ISG15*	*COL11A1, ALDH1L2, VCAN, HECTD2*
GO:0030705 cytoskeleton-dependent intracellular transport	1.51E-04	1.45E-02	*UCHL1*	*TTC21B, KIF23, SSX2IP*
GO:0048565 digestive tract development	2.54E-04	2.06E-02	*ASS1*	*KIT, HES1, SFRP2*
GO:0097367 carbohydrate derivative binding	6.08E-04	3.46E-02	*SEPT6, LIMK1, ASS1, KHK, HP, OAS2*	*KIT, VCAN, CHEK1, DNAH11, KIF23, HELQ, NRK*
GO:0007155 cell adhesion	3.18E-03	6.50E-02	*ASS1, APOD*	*KIT, VCAN, HES1, SFRP2, CDH11, SSX2IP, PCDH17*
GO:0015485 cholesterol binding	3.18E-03	6.50E-02	*APOD, LXRA*	
GO:0030199 collagen fibril organization	3.34E-03	6.50E-02		*COL11A1, SFRP2*
GO:0006508 proteolysis	1.81E-02	1.14E-01	*CST6, CTSD, LXRA, ISG15, UCHL1, HP*	*LXN, SFRP2, HECTD2*
GO:0045444 fat cell differentiation	9.75E-03	1.14E-01		*NR4A2, HES1, SFRP2*
GO:0048638 regulation of developmental growth	2.48E-02	1.26E-01	*LIMK1, RNPEPL1*	*NRK*
GO:0034332 adherens junction organization	2.66E-02	1.26E-01	*APOD*	*CDH11*
GO:0031397 negative regulation of protein ubiquitination	3.22E-02	1.32E-01	*LIMK1, ISG15*	
GO:0032496 response to lipopolysaccharide	3.31E-02	1.32E-01	*MAOB, LXRA, ASS1*	
GO:0042692 muscle cell differentiation	3.74E-02	1.32E-01	*UCHL1*	*KIT, HES1, SFRP2*
GO:0005509 calcium ion binding	4.13E-02	1.76E-01		*PCDH17, VCAN, CDH11*
GO:0030036 actin cytoskeleton organization	4.51E-02	2.39E-01	*LIMK1*	*FRMD6, KIF23*
Pathway				
R-SSC-8866427 VLDLR internalization and degradation	4.04E-03	2.64E-02	*LXRA, AP2B1*	
ssc04390 hippo signaling pathway	4.13E-03	3.29E-02		*PPP1R11, FRMD6*
R-SSC-73923 lipid digestion, mobilization, and transport	1.42E-03	4.43E-02	*HSL, LXRA, AP2B1*	
R-SSC-174824 lipoprotein metabolism	7.15E-03	1.05E-01	*LXRA, AP2B1*	
R-SSC-1442490 collagen degradation	8.88E-03	1.14E-01	*CTSD*	*COL11A1*
R-SSC-804914 transport of fatty acids	1.91E-02	1.16E-01	*APOD*	
ssc04910 insulin signaling pathway	3.78E-02	1.37E-01	*EXOC7, HSL*	
ssc00590 arachidonic acid metabolism	3.56E-02	1.37E-01	*GPX3*	
R-SSC-5627117 Rho GTPases Activate ROCKs	3.79E-02	1.37E-01	*LIMK1*	

Functional analysis performed in Kobas 3.0 and Panther Gene List

### qPCR results

**Figure 1:**
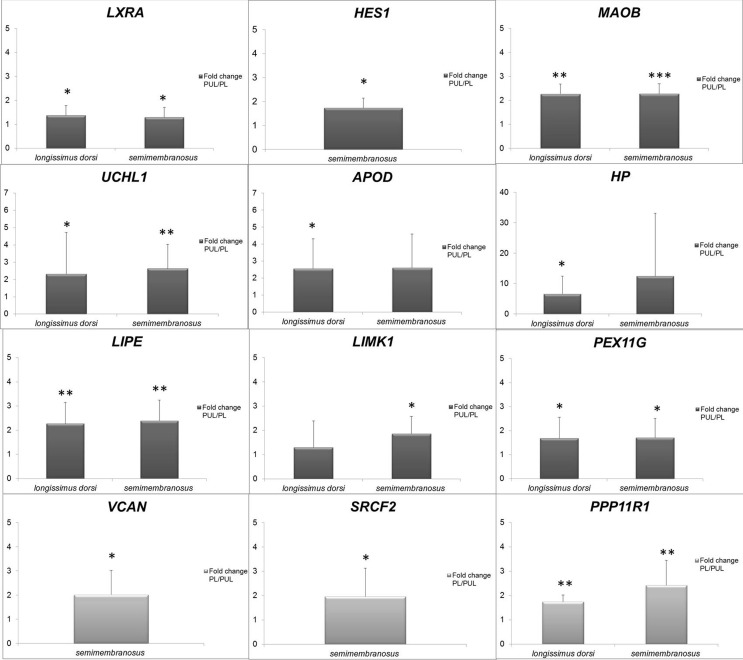
Relative quantity of mRNA shown as fold change values between analyzed pig muscles (*semimembranosus* and *longissimus dorsi*). The brighter bars show FC values based on the PL pig group as reference and the darker bars show FC values with PUL as reference. The genes of interest were normalized by two endogenous control genes, *OAZ1* and *RPL27.* *P ≤ 0.05, **P ≤ 0.01, ***P ≤ 0.001.

Validation by qPCR is commonly used to confirm RNA-seq results (Piórkowska *et al.*, 2016, Ropka-Molik *et al.*, 2014b), and this was done here for 17 differentially expressed genes. The comparison between RNA-seq and qPCR results was performed by Pearson’s correlation test. The lowest result was observed for the *LIPE* gene (*r*=0.57, *P* ≤ 0.05). Figure 1 presents the fold change of expression levels for the most important genes. Genes encoding proteins associated with lipid homeostasis were overexpressed in PUL muscle (Table 3), while those involved in cell adhesion, such as *VCAN* and *HES1* (Table 4), showed higher transcript levels in PL muscle. The fold change of gene expression values between PUL and PL for RNA-seq (FPKM) and qPCR (RQ of mRNA) results are uploaded for consultation at goo.gl/eaXXcO.

### Correlation of gene expression and pig traits

**Figure 2:**
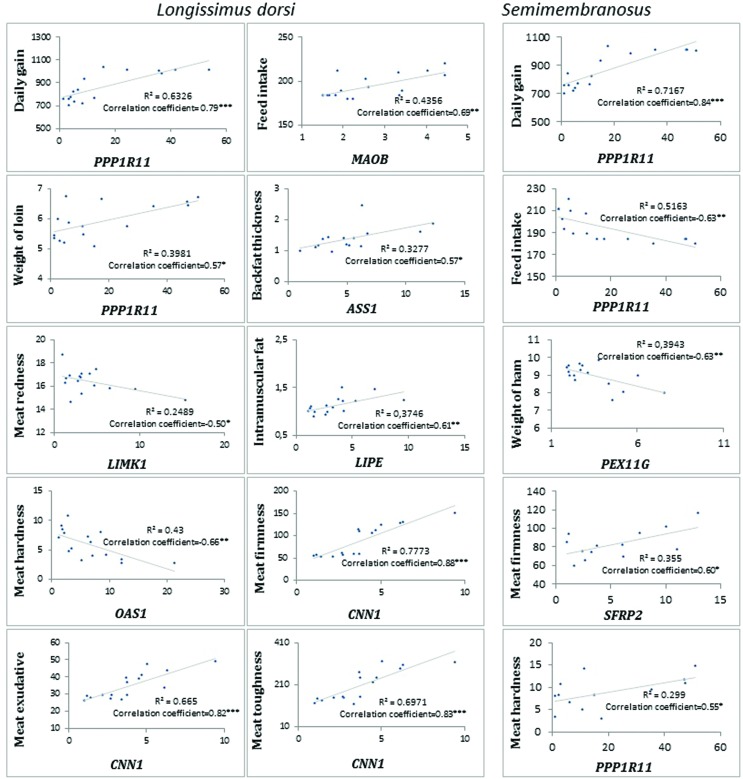
Regression of genes with differential expression in *longissimus dorsi* and *semimembranosus* muscles against important pig traits. Gene expression is presented as normalized FPKM. Unit: daily gain (g), feed intake (kg), backfat thickness (cm), weight of loin (kg), weight of ham (kg), meat redness (A*), intramuscular fat (%), meat firmness (N/mm/s), meat hardness (N/mm/s), meat exudation (cm2). *P ≤ 0.05, **P ≤ 0.01, ***P ≤ 0.001.

The correlation analysis between porcine phenotypic traits and FPKM values revealed many interesting results. *PPP11R1* and *CNN1* expressions in *longissimus dorsi* were positively correlated with daily gains (*r*=0.80, corrected *P*<0.01 and *r*=0.68, corrected *P*<0.05, respectively). In turn, *MAOB* expression in both muscles was positively associated with feed intake (LD *r*=0.66, corrected *P*<0.05 and S *r*=0.66, corrected *P*<0.05). Furthermore, *LIMK1* expression in *semimembranosus* was negatively related to meat percentage (*r*= -0.68, corrected *P*<0.05), and highly positive correlation results between *PPP11R1, PVALB, CNN1* expressions in LD and meat texture parameters were obtained. The expression of *UCHL1* in LD muscle showed a negative association with water exudation (Tables S4 and S5, Figure 2).

## Discussion

The breeding strategy that has been focused on lean pork led to a reduction of IMF percentage and meat tenderness. Therefore, searching for genetic markers for these traits seems a promising strategy. The new possibilities using innovative molecular techniques enable to indicate physiological processes involved in the shaping of farm animal traits. The present study attempted to identify genes and metabolic pathways that may influence meat quality and fat traits in pigs.

### Genes overexpressed in Pulawska pigs

Because the PUL breed has not been under selection pressure focused on high meat content in the carcass, the PUL pigs used in the present study were characterized by high backfat mass and pork quality, including good meat texture parameters and low water exudation. As in the other indigenous breeds, the PUL showed poor growth traits included daily gain (200 g lower than PL), feed conversion (0.3 kg/kg higher than PL) and low mass of the most important carcass cuts.

The transcriptomic analysis of PUL muscles showed that genes associated with fat content and lipid metabolism revealed increased expression (*LIPE, APOD, LXRA, ENSSSCG00000028753*, *AP2B1, GPX3* and *ASS1*). The *LIPE* gene, which encodes a hormone-sensitive lipase, plays a role in triacylglycerol biosynthesis. The main function of LIPE is to mobilize stored fats by hydrolyzing the first fatty acid from a triacylglycerol molecule (Holm *et al.*, 1994). The *LIPE* gene was previously analyzed regarding fatty acid composition in pigs. Xue *et al.* (2015) showed that *LIPE* (*HSL*) c.442 G > A polymorphism affected the content of C12:0 and C14:0 fatty acids (*P*<0.05). The correlation analysis carried out in the present study identified trends indicating that *LIPE* expression in the *longissimus dorsi* muscle is positively related to IMF and backfat thickness values (but after Bonferroni correction the results were not significant). These findings should stimulate the search for genetic markers for fat content in the *LIPE* gene, which could be a good direction for further research.

The *APOD* and *LXRA* (*NR1H3*) genes encode proteins involved in the LXR/RXR activation pathway, and both have the ability to bind cholesterol. APOD promotes the conversion of HDL to LDL. Desai *et al.* (2002) found in Africans a few missense mutations, *Phe36Val*, *Tyr108Cys* and *Thr158Lys,* that were associated with elevated triglyceride levels and reduced HDL-cholesterol in plasma. Whereas, LXRA plays a role in lipid homeostasis by regulating the expression of genes involved in controling feed intake and cholesterol efflux (Christoffolete *et al.*, 2010). In pigs, Yu *et al.* (2006) identified that *LXRA HpyCH4*III polymorphism was significantly associated with the total lipid content of Berkshire and Yorkshire carcasses.

In turn, the *ENSSSCG00000028753 (CYP4F2* ortholog*)*, *GPX3* and *ASS1* genes were described by Ponsuksili *et al.* (2011) as having an effect on the ‘fat area’ in pigs. Glutathione peroxidase 3 (GPX3) containing a selenocysteine residue at its active site protects the cell from oxidative damage by the reduction of lipid peroxides (Takahashi *et al.*, 1987). A previous study showed that GPX3 levels in plasma were highly correlated with the triacylglycerides/HDL-cholesterol index and body mass in humans (Baez-Duarte *et al.*, 2012). In the present study, pigs showing higher *GPX3* expression in *semimembranosus* muscle have remained longer in the test conducted by Pig Station, which meant that they took more time to reach the weight of 100 kg (correlation with slaughter age, *r*=0.76, corrected *P*-value<0.01). This suggests that genes associated with lipid deposition are negatively correlated with pig weight gains. In turn, *CYP4F2* encodes the leukotriene-B(4) omega-hydroxylase 1 protein, which is a member of the cytochrome 450 superfamily. This hydrolase plays a role in the conversion of arachidonic acid to 20-hydroxyeicosatetraenoic acid (20-HETE), and thereby, CYP4F2 reduces the fatty acid metabolite content and also is a preventing factor of lipotoxicity in fatty liver disease by regulation of the fatty acid metabolism (Hardwick *et al.*, 2010). The above observations indicate a similar regulation involved in fat deposition and the appetite and satiety processes both in humans and pigs. This confirms the theory that pig is suitable for modeling of metabolic processes associated with human obesity, as previously suggested by Spurlock and Gabler (2008).

On the other hand, the PUL pigs used in the present study showed good meat tenderness and low water exudation. The RNA-seq analysis showed that in PUL muscles, genes (*CST6, CTSD*, *ISG15, UCHL1* and *HP*) involved in proteolysis process were overexpressed. The correlation analysis revealed that *UCHL1* expression in the *longissimus dorsi* muscle was negatively related to meat exudation (*r*= -0.66, corrected *P*<0.05). The *UCHL1* gene encodes ubiquitin C-terminal hydrolase L1 that hydrolyzes a peptide bond at the C-terminal glycine of ubiquitin. In the mouse model, it was observed that elevation of UCHL1 in fibroblasts was associated with spinal muscular atrophy (Hsu *et al.*, 2010). However, the literature did not provide any information about the function of UCHL1 in muscle. The *HP* gene encodes haptoglobin, which is a circulating acute-phase protein having an anti-oxidant function. In humans, *HP* expression is induced in white adipose tissue, which is reflected in the increased plasma levels of the glycoprotein found in obese subjects (Chiellini *et al.*, 2004). In the present study, it was observed that *HP* expression in the *semimembranosus* was negatively correlated with pH estimated 24 h after slaughter (corrected *P*<0.05). Thus, it could be closely related to the proteolysis process, which is activated *post mortem* in response to a decrease in pH, which then determines meat tenderness (Huff-Lonergan *et al.*, 2010). However, no significant correlation of *HP* expression and meat texture parameters was observed in this study. The HP and UCHL1 functions are not exactly clear, but their increased expression in muscle tissue of pigs having high meat quality seems to be interesting. Therefore, they should be considered in further research.

The differentially expressed isoform analysis showed that in PUL muscles the seventh isoform of the *SEPT6* gene was up-regulated. This *SEPT6-007* isoform has a different length of the UTR region than the principal protein. *SEPT6* encodes septin 6 that interacts with Rho, regulating the actin cytoskeleton in the GTPases pathway (Mostowy and Cossart, 2012). In addition, SEPT6 plays a role in cytokinesis, contributing to cell proliferation, and this could be associated with its influence on pork texture parameters.

### Genes overexpressed in Polish Landrace pigs

These pig breed belong to the white pigs, having good growth traits, but its pork, as a consequence of breeding, has low quality. In the present study, the PL pigs presented extremely low meat quality, which allowed for a capture of genes associated with meat tenderness. Usually, the quality of PL pork is not so low (Ropka-Molik *et al.*, 2016). Nevertheless, this parameter is not considered during selection, which could lead to additional deterioration of PL pork quality. Therefore, the aim of this research was to reveal candidate genes for pork quality traits.

During RNA-seq analysis, it was found that genes involved in actin filament building, collagen fibril organization and focal adhesion were overexpressed in PL muscles. Two up-regulated genes were found as playing a role in an interesting physiological pathway, such as Hippo signaling (*PPP1R11, FRMD6*). Hippo signaling regulates organ growth in Drosophila and vertebrates, controling the specification, differentiation and proliferation of cells. *PPP1R11* encodes phosphatase 1 regulatory (inhibitor) subunit 11 (PPP1R11) that activates the YAP/TAZ complex, the main complex in Hippo signaling participating in apoptosis and cell proliferation (Halder and Johnson, 2011). In muscle, the mature fibers do not have the ability to proliferate, but the discovery of the myosatellite cells changed the view towards the emergence of new muscle fibers postnatally (Blaauw and Reggiani, 2014). The myosatellite cells have a multipotent character. They also can differentiate in the postnatal stage, thereby having an effect on myofibrillar network organization, contributing to the shaping of meat texture and water capacity (Bhat and Fayaz, 2011), and also growth traits. In the present correlation analysis, *PPP1R11* expression in LD muscle was highly negatively associated with tenderness (highly positive correlation with firmness value *r*=0.76, corrected *P*<0.01 and toughness value *r*=0.77, corrected *P*<0.01). In addition, the expression level measured in both investigated muscles was positively related with daily gain (LD *r*=0.80, S *r*=0.85, corrected *P*<0.01). It could be suggested that PPP1R11 plays some role in muscle fiber proliferation that occurs postnatally. Moreover, PPP1R11 is probably negatively associated with glucagon storage. Qiu *et al.* (2014) observed the overexpression of *PPP1R11* in muscles of patients with diabetes mellitus type 1. Similarly Yang *et al.* (2000) identified increased *PPP1R11* expression in skeletal muscles of insulin-sensitive Pima Indians. In turn, Kettunen *et al.* (2012) found that a single nucleotide polymorphism in *PPP1R11* was associated with VLDL particle concentration in plasma of Finnish cohorts. On the other hand, the present correlation analysis showed a highly significant positive association between *PPP1R11* expression in LD muscle and meat exudation measured also in *longissimus dorsi* (*r*=0.82, corrected *P*<0.01). Our observation confirmed the previous studies that reported that water loss was positively correlated with IIb type fibers and negatively with I and IIa type fibers (Ryu and Kim, 2005; Wojtysiak and Poltowicz, 2014) showing a much higher percentage of IIb fiber in the PL skeletal muscles compared to the PUL breed. In view of the above evidence, the *PPP1R11* gene is of interest in many contexts, both in its involvement in proliferation processes, glucagon storage, and influence on meat texture. Therefore, *PPP1R11* should be widely investigated when searching for genetic markers for meat quality.

A highly significant correlation was also identified for *CNN1* expression in *longissimus dorsi*. Increased *CNN1* expression was positive associated with daily gain (*r*=0.68, corrected *P*<0.05), water exudation (*r*=0.82, corrected *P*<0.01), and negatively with tenderness (firmness *r*=0.81, corrected *P*<0.01, toughness *r*=0.83, corrected *P*<0.01). *CNN1* encodes Calponin 1, which is a thin filament-associated protein contributing to the modulation and regulation of smooth muscle contraction. Calponin 1 is capable of binding to actin, calmodulin, troponin C and tropomyosin. The interaction with actin inhibits actomyosin Mg-ATPase activity (Samaha *et al.*, 1996). The *CNN1* gene should be further analyzed in the context of effect on pork quality and growth traits.

On the other hand, in skeletal muscle of PL pigs, genes involved in calcium ion binding (*PVALB, CIB2*, *PCDH17* and *CDH11*) were overexpressed. Ca^2+^ ions play a key role in the quality of pork through determining calpain activity (Lian *et al.*, 2013), which is important in the tenderization process. *CIB2* encodes calcium and integrin binding family member 2 protein (CIB2) that binds a novel integrin, α7Bβ1D. The absence of this integrin in muscle tissue results in myopathy, both in mice and humans (Häger *et al.*, 2008). In turn, the protein encoded by *PVALB* is involved in the relaxation of muscle by rapidly sequestering calcium from the sarcoplasm of the cell (Mutryn *et al.*, 2015). In the present study, a high correlation was observed between *PVALB* expression and meat texture parameters (firmness *r*=0.71, corrected *P*<0.05, toughness *r*=0.68 corrected *P*<0.05) in LD muscle, which could be associated with its role in the calcium efflux process. In turn, the cadherins PCDH17 and CDH11 promote the fusion of mononuclear myoblast cells into polynuclear myotubes, which is one of the essential steps in myogenesis (Waibler and Starzinski-Powitz, 2002). Thus, these genes could influence pork texture parameters by determining muscle fiber development.

## Conclusions

The study presents a gene cluster (*PPP1R11, SFRP2, CIB2, PVALB, UCHL1* and *CNN1*) that is probably associated with meat quality via regulating cell proliferation and differentiation, and calcium binding in muscles. Moreover, we propose a number of genes as candidates for fat content in pigs (*LIPE, LXRA, HP*), which were previously investigated in terms of human obesity. These candidate genes should be analyzed in future association studies, aiming at identifying genetic markers.

## Supplementary material

The following online material is available for this article:

Table S1 - Overall statistics and read annotations obtained for each library.

Table S2 - Primer and TaqMan probes used in validation.

Table S3 - Genes differentially expressed in *semimembranosus* muscle of Polish Landrace pigs.

Table S4 - Pearson correlation coefficient for DEGs in *longissimus dorsi* and pig production traits.

Table S5 - Correlation coefficient for DEGs in *semimembranosus* and pig production traits.
