# Supplementary material for: A comprehensive transcriptome analysis of skeletal muscles in two Polish pig breeds differing in fat and meat quality traits
Source: Genet Mol Biol. 2018 Jan-Mar;41(1):125–36. doi: 10.1590/1678-4685-GMB-2016-0101 (PMC5901489; doi:10.1590/1678-4685-GMB-2016-0101)
Supplement: Supplementary file 1 [file 1415-4757-GMB-41-01-2016-0101-s001.pdf]

Supplementary Material to “A comprehensive transcriptome analysis of skeletal muscles in two Polish pig breeds differing in fat and meat quality traits”

Table S1 - Overall statistics and read annotations obtained for each library.

Sample	PULS1	PULS2	PULS3	PULS4	PULS5	PULS6	PULS7	PULS8
Indexes/pooling (lane)	AR005/lane2*	AR009/lane2	AR011/lane2	AR015/lane2	AR022/lane2	AR025/lane2	AR012/lane2	AR014/lane2
Remaining reads %	99.0	99.2	99.2	99.1	99.0	99.3	99.4	99.2
Exonic rate	63.2	66.3	69.1	66.2	66.2	65.1	66.2	69.4
Intronic rate	8.1	7.5	5.9	7.6	9.3	7.8	8.3	6.3
Base mismatch rate	0.42	0.40	0.47	0.49	0.41	0.47	0.47	0.46
Uniquely mapped reads %	82.43	83.07	79.44	81.28	84.01	79.82	81.45	79.14
% of reads mapped to multiple loci	7.68	8.19	8.05	7.48	7.82	6.89	6.61	7.07
Sample	PULL1	PULL2	PULL3	PULL4	PULL5	PULL6	PULL7	PULL8
Indexes/pooling (lane)	AR005/lane1*	AR009/lane1	AR011/lane1	AR015/lane1	AR022/lane1	AR025/lane1	AR012/lane1	AR014/lane1
Remaining reads %	99.3	99.3	99.3	99.3	99.3	99.1	99.2	99.2
Exonic rate %	63.4	63.0	68.6	65.7	68.4	63.0	66.6	66.3
Intronic rate %	9.9	9.3	6.5	8.6	6.9	8.2	8.7	7.5
Base mismatch rate %	0.31	0.35	0.34	0.37	0.42	0.39	0.42	0.34
Uniquely mapped reads %	83.6	83.3	80.8	82.9	79.9	76.7	80.8	82.0
% of reads mapped to multiple loci	9.3	8.6	7.7	7.6	8.4	7.0	7.7	6.8
Sample	PLL1	PLL2	PLL3	PLL4	PLL5	PLL6	PLL7	PLL8
Indexes/pooling (lane)	AR006/lane1*	AR007/lane1	AR008/lane1	AR010/lane1	AR020/lane1	AR027/lane1	AR016/lane1	AR023/lane1
Remaining reads %	99.3	99.0	99.3	99.3	99.3	99.1	99.2	99.2
Exonic rate %	66.3	60.7	64.1	65.1	67.6	70.2	65.5	63.7
Intronic rate %	8.0	10.3	8.7	7.5	7.5	7.3	8.4	7.8
Base mismatch rate %	0.41	0.54	0.44	0.50	0.43	0.57	0.42	0.43
Uniquely mapped reads %	83.5	67.6	82.8	80.6	83.5	73.1	82.6	82.9
% of reads mapped to multiple loci	7.8	6.7	7.5	7.6	7.05	8.8	7.1	7.0
Sample	PLS1	PLS2	PLS3	PLS4	PLS5	PLS6	PLS7	PLS8
Indexes/pooling (lane)	AR006/lane2*	AR007/lane2	AR008/lane2	AR010/lane2	AR020/lane2	AR027/lane2	AR016/lane2	AR023/lane2

Sample	PLS1	PLS2	PLS3	PLS4	PLS5	PLS6	PLS7	PLS8
Remaining reads %	99.3	99.3	99.3	99.3	99.2	99.1	99.3	98.9
Exonic rate %	66.9	66.6	67.2	67.9	70.4	67.3	66.6	64.7
Intronic rate %	6.9	8.2	6.9	6.3	7.1	6.5	9.4	7.4
Base mismatch rate %	0.40	0.38	0.40	0.39	0.47	0.40	0.37	0.35
Uniquely mapped reads %	81.1	81.3	81.6	83.6	79.4	82.1	82.4	81.2
% of reads mapped to multiple loci	7.01	7.7	7.3	7.2	8.2	7.03	7.52	7.43

Abbreviation: PULS and PLS – ham (semimembranosus) samples of Puławska and Polish Landrace pigs, PULL and PLL loin (longissimus dorsi) samples of Puławska and Polish Landrace pigs, * All loin samples were analysed on in four technical replicates in 1,3,5,7 lanes; in turn loin samples in 2, 4, 6,8 lanes
