# Supplementary material for: A comprehensive transcriptome analysis of skeletal muscles in two Polish pig breeds differing in fat and meat quality traits
Source: Genet Mol Biol. 2018 Jan-Mar;41(1):125–36. doi: 10.1590/1678-4685-GMB-2016-0101 (PMC5901489; doi:10.1590/1678-4685-GMB-2016-0101)
Supplement: Supplementary file 2 [file 1415-4757-GMB-41-01-2016-0101-s002.pdf]

Supplementary Material to "A comprehensive transcriptome analysis of skeletal muscles in two Polish pig breeds differing in fat and meat quality traits"

Table S2 - Primer and TaqMan probes used in validation.

Gene		Primers and Probe or Taqman Gene Expression Assay ID	Amplicon length [bp]	Label	Reference sequences accesion numbers	Exon Boundary
PPP1R11	protein phosphatase 1 regulatory inhibitor subunit 11	Ss03390641_m1	73	FAM	NM_001123166.1	1-2
		Ss03390642_g1	72	FAM	NM_001123166	2-3
		TCACTGAGACAACGGTTACC<br>GGAAGGTCCCAGCAAATCTTC	95	EvaGreen	ENSSSCG00000028347	2-3
		CGGAAGCCAGAGAAAAAGGT<br>TATGACCACAGCCCTCCTCT	159	EvaGreen	ENSSSCG00000028347	3-4
UCHL1	ubiquitin carboxyl-terminal esterase L1 (ubiquitin thiolesterase)	Ss03381306_u1	131	FAM	NM_213763	2-3
HES1	hes family bHLH transcription factor 1	Ss04321631_g1	75	FAM	BX930390.1	2-3
MAOB	monoamine oxidase B	Ss03378378_u1	74	VIC	NM_001195231	15-15
SFRP2	secreted frizzled-related protein 2	Ss04327215_m1	99	VIC	NM_001244395.1	1-2
VCAN	versican	Ss04323138_m1	67	VIC	NM_001206429.1	3-4
HP	haptoglobin	Ss03393371_u1	90	FAM	NM_214000.2	5-5
OAS2	2'-5'-oligoadenylate synthetase 2, 69/71kDa	Ss03392009_m1	65	FAM	NM_213577.1	4-5
PVALB	parvalbumin	Ss04247063_m1	86	FAM	NM_001190157.1	3-4
GPX3	glutathione peroxidase 3 (plasma)	Ss03387487_u1	80	FAM	NM_001115155.1	5-5
CNN1	calponin 1, basic, smooth muscle	Ss03392449_g1	83	VIC	NM_213878.1	4-5
ASS1	argininosuccinate synthase 1	Ss03373750_m1	65	VIC	AK232885.1	12-13
APOD	Apolipoprotein D	CTCCTTGGAGTGGCTGAGG<br>GCTTGGATGCAACTTCCCTT	152	EvaGreen	ENSSSCG00000011831	2-3

Gene		Primers and Probe<br>or<br>Taqman Gene Expression Assay ID	Amplicon length [bp]	Label	Reference sequences accesion numbers	Exon Boundary
PEX11G	Peroxisomal biogenesis factor 11 gamma	CCAGCTCTACTACCCCTGTG<br>CGTCTCAGCTTCAGGACCAT	158	EvaGreen	ENSSSCG00000013569	2-3
LIMK1	LIM domain kinase 1	TGCAAGAAGGACTACTGGGC<br>CCAGAGTGTAGGTGTCCCC	165	EvaGreen	ENSSSCG00000030378	2-3
LXRA (NR1H3)	nuclear receptor subfamily 1, group H, member 3	Ss03389237_g1	101	VIC	NM_001101814.1	5-6
LIPE	lipase E, hormone sensitive type	Ss03383966_u1	96	FAM	NM_214315.1	9-9
OAZ1	ornithine decarboxylase antizyme 1<br>**housekeeping gene**	Ss03387505_u1	77	VIC	NM_001122994.1	5-5
RPL27	60S ribosomal protein L27<br>**housekeeping gene**	5`NED – CGGTCATCGTAAAGAA<br>CGCTACTCCGGACGCAAA<br>GGTCTGAGGTGCCATCATCA	55	NED	ENSSSCG00000025507	1-2
