# Supplementary material for: A comprehensive transcriptome analysis of skeletal muscles in two Polish pig breeds differing in fat and meat quality traits
Source: Genet Mol Biol. 2018 Jan-Mar;41(1):125–36. doi: 10.1590/1678-4685-GMB-2016-0101 (PMC5901489; doi:10.1590/1678-4685-GMB-2016-0101)
Supplement: Supplementary file 3 [file 1415-4757-GMB-41-01-2016-0101-s003.pdf]

Supplementary Material to "A comprehensive transcriptome analysis of skeletal muscles in two Polish pig breeds differing in fat and meat quality traits"

Table S3. – Genes ≥ ±1.5– fold (up-regulated and down-regulated) differentially expressed in *semimembranosus* muscle of Polish Landrace pigs.

Gene		Ensemble ID	DESeq		edgeR		baySeq FDR	Validation qPCR PC[1]	
symbol	name		FC	Ad p-value	FC	FDR		r	P
PPP1R11	Protein phosphatase 1, regulatory (inhibitor) subunit 11	ENSSSCG00000028347	2.75	***	6.16	***	***	0.63	≤0.05
NG	Similar to E3 ubiquitin-protein ligase (HECTD2)	ENSSSCG00000020912	2.01	**	2.49	*	**		
PCDH17	Protocadherin 17	ENSSSCG00000009446	2.00	**	2.45	**	***		
NG	Similar to Nik related kinase (NRK)	ENSSSCG00000030276	1.98	*	2.83	*	**		
HES1	Hairy and enhancer of split 1	ENSSSCG00000026362	1.95	**	2.47	*	**	0.91	≤0.001
SFRP2	Secreted frizzled-related protein 2	ENSSSCG00000009004	1.94	*	2.73	*	***	0.87	≤0.001
VCAN	Versican	ENSSSCG00000014136	1.93	*	3.11	*	*	0.82	≤0.001
NR4A2	Nuclear receptor subfamily 4, group A, member 2	ENSSSCG00000015871	1.88	*	3.22	*	*		
CHEK1	Checkpoint kinase 1	ENSSSCG00000015215	1.82	*	2.21	0.057	**		
MYZAP	Myocardial zonula adherens protein	ENSSSCG00000004598	1.81	*	2.19	0.052	*		
LXN	Latexin	ENSSSCG00000020984	1.80	*	2.19	*	***		
COL11A1	Collagen, type XI, alpha 1	ENSSSCG00000006857	1.78	0.051	3.43	*	*		
SSX2IP	Synovial sarcoma, X breakpoint 2 interacting protein	ENSSSCG00000003757	1.78	*	2.20	0.063	*		
FRMD6	FERM domain containing 6	ENSSSCG00000005027	1.74	*	2.09	0.064	**		
NUF2	NDC80 kinetochore complex component	ENSSSCG00000006333	1.72	*	2.03	0.076	*		
KIF23	Kinesin family member 23	ENSSSCG00000004969	1.72	*	1.97	0.078	**		
NG	Similar to transmembrane protein 106C (*TMEM106C*)	ENSSSCG00000028397	1.71	*	1.98	0.067	*		
DNAH11	Dynein, axonemal, heavy chain 11	ENSSSCG00000015379	1.71	*	1.96	0.082	*		

Gene		Ensemble ID	DESeq		edgeR		baySeq	Validation qPCR PC[1]	
symbol	name		FC	Ad p-value	FC	FDR	FDR	r	P
KIT	V-kit Hardy-Zuckerman 4 feline sarcoma viral oncogene	ENSSSCG00000008842	1.69	*	1.94	0.1	*		
ZNF300	Zinc finger protein 300	ENSSSCG00000021371	1.64	*	1.85	0.013	**		
NG	Similar to trichoplein, keratin filament binding (*TCHP*)	ENSSSCG00000009926	1.64	*	1.85	0.086	*		
CDH11	Cadherin 11, type 2, OB-cadherin	ENSSSCG00000002795	1.56	0.076	2.93	*	**		
NG	Similar to tetratricopeptide repeat domain 21B (*TTC21B*)	ENSSSCG00000015909	1.57	*	1.67	0.01	**		
ALDH1L2	Aldehyde dehydrogenase 1 family, member L2	ENSSSCG00000000839	1.57	**	1.65	0.066	*		
C5orf42	Chromosome 5 open reading frame 42	ENSSSCG00000016843	1.55	*	1.67	0.17	*		
CCDC25	Coiled-coil domain containing 25	ENSSSCG00000024972	1.54	***	1.59	0.052	**		
HELQ	Helicase, POLQ-like	ENSSSCG00000009236	1.53	*	1.60	0.17	*		
CXorf38	Chromosome X open reading frame	ENSSSCG00000012249	1.46	**	1.51	0.19	*		
ISG15	ISG15 ubiquitin-like modifier	ENSSSCG00000027982	-1.13	0.073	-7.71	*	***		
TRNAU1AP	tRNA selenocysteine 1 associated protein 1	ENSSSCG00000030284	-1.37	**	-1.38	0.023	**		
RGP1	Retrograde golgi transport homolog	ENSSSCG00000005320	-1.38	*	-1.40	0.29	*		
NG	Similar to PPFIA binding protein 2 (PPFIBP2)	ENSSSCG00000014598	-1.41	*	-1.43	0.26	*		
THTPA	Thiamine triphosphatase	ENSSSCG00000002016	-1.45	*	-1.48	0.19	**		
NR1H3	Nuclear receptor subfamily 1, group H, member 3 (LXRA)	ENSSSCG00000013241	-1.48	*	-1.53	0.13	**	0.64	≤0.05
MAGIX	MAGI family member, X-linked	ENSSSCG00000012295	-1.52	*	-1.57	0.11	*		
NG	Similar to DnaJ homolog subfamily C member 8 (DNAJC8)	ENSSSCG00000028809	-1.54	**	-1.58	0.052	**		
NG	Similar to dual specificity phosphatase 13 (DUSP13)	ENSSSCG00000029128	-1.55	*	-1.65	0.17	*		
GPX3	Glutathione peroxidase 3	ENSSSCG00000017092	-1.57	0.022	-4.07	*	***	0.98	≤0.001
AP2B1	Adaptor-related protein complex 2, beta 1 subunit	ENSSSCG00000024434	-1.57	**	-1.61	0.052	**		
RNPEPL1	Arginyl aminopeptidase (aminopeptidase B)-like 1	ENSSSCG00000016367	-1.59	*	-1.70	0.064	*		
KHK	Ketohexokinase (fructokinase)	ENSSSCG00000008556	-1.59	*	-1.73	0.110	*		
SLC38A3	Solute carrier family 38, member 3	ENSSSCG00000011397	-1.60	*	-1.73	0.086	**		
CST6	Cystatin E/M	ENSSSCG00000012960	-1.60	*	-1.68	0.063	**		
NG	Cathepsin D (CTSD)	ENSSSCG00000026418	-1.60	*	-1.69	0.076	*		

Gene		Ensemble ID	DESeq		edgeR		baySeq	Validation qPCR PC[1]	
symbol	name		FC	Ad p-value	FC	FDR	FDR	r	P
EXOC7	Exocyst complex component 7	ENSSSCG00000017190	-1.66	**	-1.77	*	**		
ASS1	Argininosuccinate synthase 1	ENSSSCG00000005701	-1.68	0.12	-6.07	*	***	0.98	≤0.001
LRRC30	Leucine rich repeat containing 30	ENSSSCG00000023672	-1.75	*	-2.00	0.073	***		
MAOB	Monoamine oxidase B	ENSSSCG00000023215	-1.77	**	-1.96	*	*		
UCHL1	Ubiquitin carboxyl-terminal esterase L1	ENSSSCG00000022945	-1.84	*	-2.43	0.056	*	0.81	≤0.01
PEX11G	Peroxisomal biogenesis factor 11 gamma	ENSSSCG00000013569	-1.86	**	-2.07	*	***	0.70	≤0.01
LIPE	Lipase, hormone-sensitive (HSL)	ENSSSCG00000003018	-1.87	*	-2.51	*	**	0.57	≤0.05
LIMK1	LIM domain kinase 1	ENSSSCG00000030378	-1.89	*	-2.36	*	**	0.85	≤0.01
OAS2	Helicase, POLQ-like	ENSSSCG00000009881	-1.93	*	-3.36	*	***	0.90	≤0.001
HP	Haptoglobin	ENSSSCG00000002749	-2.10	**	-11.18	***	***	0.99	≤0.001
NG	Similar to SWI/SNF related, matrix associated, actin dependent	ENSSSCG00000022002	-2.14	**	-2.91	**	***		
APOD	Apolipoprotein D	ENSSSCG00000011831	-2.26	***	-2.67	***	***	0.89	≤0.001
SEPT6-007	6-isoform of septin 6	ENSSSCT00000013798	-1.95	**	-2.24	**	***		

Abbreviation: Ad *P*-value - adjusted *P*-value, FDR- false discovery rate; rate of type I errors, FC- fold change, PL –Polish Landrace pigs, NG – novel gene according to Ensembl browser
[1]The Pearson Correlation Coefficients had *P*-value at least <0.05, *P*-values of DESeq, edgeR and baySeq methods****P*<0.001, ***P*<0.01, **P*<0.05.
