# Supplementary material for: A comprehensive transcriptome analysis of skeletal muscles in two Polish pig breeds differing in fat and meat quality traits
Source: Genet Mol Biol. 2018 Jan-Mar;41(1):125–36. doi: 10.1590/1678-4685-GMB-2016-0101 (PMC5901489; doi:10.1590/1678-4685-GMB-2016-0101)
Supplement: Supplementary file 4 [file 1415-4757-GMB-41-01-2016-0101-s004.pdf]

Supplementary Material to "A comprehensive transcriptome analysis of skeletal muscles in two Polish pig breeds differing in fat and meat quality traits"

Table S4 - Pearson correlation coefficient r between FPKMs of DEGs in *longissimus dorsi* and pig production traits.

Trait	Differentially expressed genes																
	ASS1	*UCHL1*	*LIPE*	*HES1*	*LXRA*	*MAOB*	*SCRF2*	*VCAN*	*APOD*	*LIMK1*	*PEX11G*	*PPP11R1*	*HP*	*OAS2*	*PVALB*	*CNN1*	*GPX3*
**Fattening traits**																	
age at slaughter (day)	0.49	0.70**	0.05	-0.19	0.51*	0.57*	0.01	-0.16	0.19	0.53*	0.49*	-0.32	0.43	-0.67**	-0.34	-0.45	0.28
daily gain (g/kg)	-0.41	-0.58*	-0.39	0.01	-0.41	-0.61*	0.26	0.49	-0.18	-0.40	-0.56*	0.80***	-0.35	-0.54*	0.54*	0.68**	-0.24
feed gain ration (g/day)	0.40	0.47	0.29	-0.12	0.41	0.69**	-0.31	-0.57*	0.02	0.32	0.45	-0.63**	0.18	0.44	-0.46	-0.47	0.05
feed intake (kg)	0.62*	0.54*	0.34	-0.07	0.32	0.66**	-0.30	-0.49	-0.01	0.57*	0.43	-0.59*	0.23	0.48	-0.56*	-0.48	0.02
**Slaughter traits**																	
weight of loin (kg)	0.05	-0.03	-0.41	0.03	-0.23	-0.26	0.21	0.51	-0.14	0.16	-0.19	0.57*	-0.15	-0.01	0.08	0.29	-0.17
backfat thickness (cm)	0.57*	0.31	0.55*	0.30	0.18	0.28	-0.09	-0.21	0.27	0.35	0.37	-0.44	0.44	0.40	-0.53*	-0.43	0.29
loin eye area (cm2)	-0.41	-0.2	-0.49	-0.24	-0.25	-0.27	0.22	0.57*	-0.19	-0.27	-0.27	0.28	-0.38	-0.19	0.06	0.27	-0.26
meat percentage (%)	-0.63*	-0.39	-0.55*	-0.24	-0.45	-0.51*	0.05	0,07	-0.41	-0.33	-0.53*	0.35	-0.51*	-0.31	0.58*	0.39	-0.39
Longissimus dorsi																	
meat exudation	-0.21	-0.66**	-0.30	0.49	-0.42	-0.61*	0.18	0.46	-0.28	-0.37	-0.57*	0.82***	-0.41	-0.55*	0.45	0.82***	-0.26
meat redness (MA)	-0.28	-0.30	-0.13	0.31	-0.23	-0.23	0.25	0.40	0.11	-0.50*	-0.09	0.42	-0.19	-0.21	0.38	0.50*	-0.02
Intramuscular fat (%)	0.48	0.07	0.61**	0.35	0.21	0.21	-0.01	-0.07	0.23	0.13	0.20	-0.27	0.28	0.01	-0.37	-0.18	0.22
firmness by Warner-Bratzler (N)	-0.36	-0.64**	-0.41	0.37	-0.38	-0.61*	0.24	0.46	-0.32	-0.41	-0.59*	0.76***	-0.47	-0.63**	0.71**	0.88***	-0.32
toughness by Warner-Bratzler (N)	-0.29	-0.61*	-0.31	0.41	-0.41	-0.60*	0.21	0.41	-0.30	-0.38	-0.61*	0.77***	-0.45	-0.60*	0.68**	0.83***	-0.30
harness by TPA (N)	-0.49	-0.53*	-0.17	0.06	-0.25	-0.39	-0.21	-0.11	-0.34	-0.10	-0.52*	-0.10	-0.28	-0.66**	0.28	0.16	-0.29

*- P≤0.05; **- P≤0.01, ***- P≤0.001, underlined values were significant after Bonferroni correction. The transcript levels of DEGs were showed as normalized FPKM values.
