# Supplementary material for: A comprehensive transcriptome analysis of skeletal muscles in two Polish pig breeds differing in fat and meat quality traits
Source: Genet Mol Biol. 2018 Jan-Mar;41(1):125–36. doi: 10.1590/1678-4685-GMB-2016-0101 (PMC5901489; doi:10.1590/1678-4685-GMB-2016-0101)
Supplement: Supplementary file 5 [file 1415-4757-GMB-41-01-2016-0101-s005.pdf]

Supplementary Material to “A comprehensive transcriptome analysis of skeletal muscles in two Polish pig breeds differing in fat and meat quality traits”

Table S5 - Correlation coefficient r between FPKMs of DEGs in *semimembranosus* and pig production traits.

Trait	Differentially expressed genes																
	ASS1	UCHL1	*LIPE*	*HES1*	*LXRA*	*MAOB*	*SCRF2*	*VCAN*	*APOD*	*LIMK1*	*PEX11G*	*PPP11R1*	*HP*	*OAS2*	*PVALB*	*CNN1*	*GPX3*
**Fattening traits**																	
age at slaughter	<u>0.69**</u>	0.22	0.23	-0.40	0.35	0.43	-0.33	-0.16	<u>0.80***</u>	0.26	0.40	-0.32	<u>0.75***</u>	<u>-0.67**</u>	-0.36	-0.31	<u>0.76***</u>
daily gain (g/kg)	-0.49	0.31	-0.49	0.63**	-0.38	-0.51*	0.42	0.57*	-0.54*	-0.53*	-0.57*	<u>0.85***</u>	-0.35	-0.54*	0.52*	0.70**	-0.53*
feed gain ration (g/day)	0.55*	0.33	0.32	-0.51*	0.23	0.54*	-0.46	-0.59*	0.46	0.41	0.52*	<u>-0.76***</u>	0.19	0.44	-0.31	-0.62**	0.54*
feed intake (kg)	0.48	0.24	0.28	-0.51*	0.15	<u>0.66**</u>	-0.51*	-0.55*	0.53*	0.56*	0.46	<u>-0.72**</u>	0.35	0.49	-0.36	-0.46	0.50*
**Slaughter traits**																	
weight of ham (kg)	-0.43	-0.59*	-0.39	0.36	-0.54*	-0.16	0.33	0.46	-0.23	-0.18	-0.63**	0.38	-0.30	0.23	0.26	0.61*	-0.31
Meat percentage (%)	-0.29	-0.42	-0.32	0.36	-0.41	-0.53*	0.53*	0.55*	-0.40	<u>-0.68**</u>	-0.58*	0.40	-0.19	-0.11	0.41	0.25	-0.27
Semimembranosus																	
firmness<br>by Warner-Bratzler	-0.15	-0.08	0	0.49	-0.11	-0.34	0.60*	0.35	-0.24	-0.31	-0.22	0.44	-0.22	-0.09	0.26	0.24	-0.12
harness by TPA	0	-0.21	0.49	0.43	0.50	-0.23	-0.07	-0.11	0.11	0.12	0.28	0.55*	0.26	-0.10	-0.01	0.15	-0.03
pH24	-0.34	-0.15	0.17	0.30	-0.02	-0.28	-0.10	-0.32	-0.65**	0.27	0.04	0.05	<u>-0.70**</u>	<u>-0.69**</u>	0.04	0.15	-0.46

*- P≤0.05; **- P≤0.01, ***- P≤0.001. <u>underlined values</u> were significant after Bonferroni correction. In additional LIPE gene although it was not validated by qPCR was correlated with pig traits. The transcript levels were showed as normalized FPKM values.
